# Thalamic Volumes and Functional Networks Linked With Self‐Regulation Dysfunction in Major Depressive Disorder

**DOI:** 10.1111/cns.70116

**Published:** 2024-11-10

**Authors:** Zhang Ling, He Cancan, Liu Xinyi, Fan Dandan, Zhang Haisan, Zhang Hongxing, Xie Chunming

**Affiliations:** ^1^ Department of Neurology, Affiliated ZhongDa Hospital, School of Medicine, Jiangsu Key Laboratory of Brain Science and Medicine Southeast University Nanjing Jiangsu China; ^2^ Department of Radiology The Second Affiliated Hospital of Xinxiang Medical University Xinxiang Henan China; ^3^ Xinxiang Key Laboratory of Multimodal Brain Imaging The Second Affiliated Hospital of Xinxiang Medical University Xinxiang Henan China; ^4^ Department of Psychiatry The Second Affiliated Hospital of Xinxiang Medical University Xinxiang Henan China; ^5^ Psychology School of Xinxiang Medical University Xinxiang Henan China; ^6^ Institute of Neuropsychiatry, Affiliated ZhongDa Hospital Southeast University Nanjing Jiangsu China; ^7^ The Key Laboratory of Developmental Genes and Human Disease Southeast University Nanjing Jiangsu China

**Keywords:** canonical correlation analysis, major depression disorder, network‐based statistic‐predict, self‐regulation, thalamic subnuclei

## Abstract

**Aims:**

Self‐regulation (SR) dysfunction is a crucial risk factor for major depressive disorder (MDD). However, neural substrates of SR linking MDD remain unclear.

**Methods:**

Sixty‐eight healthy controls and 75 MDD patients were recruited to complete regulatory orientation assessments with the Regulatory Focus Questionnaire (RFQ) and Regulatory Mode Questionnaire (RMQ). Nodal intra and inter‐network functional connectivity (FC) was defined as FC sum within networks of 46 thalamic subnuclei (TS) or 88 AAL brain regions, and between the two networks separately. Group‐level volumetric and functional difference were compared by two sample *t*‐tests. Pearson's correlation analysis and mediation analysis were utilized to investigate the relationship among imaging parameters and the two behaviors. Canonical correlation analysis (CCA) was conducted to explore the inter‐network FC mode of TS related to behavioral subscales. Network‐based Statistics with machine learning combining powerful brain imaging features was applied to predict individual behavioral subscales.

**Results:**

MDD patients showed no group‐level volumetric difference in 46 TS but represented significant correlation of TS volume and nodal FC with behavioral subscales. Specially, inter‐network FC of the orbital part of the right superior frontal gyrus and the left supplementary motor area mediated the correlation between RFQ/RMQ subscales and depressive severity. Furthermore, CCA identified how the two behaviors are linked via the inter‐network FC mode of TS. More crucially, thalamic functional subnetworks could predict RFQ/RMQ subscales and psychomotor retardation for MDD individuals.

**Conclusion:**

These findings provided neurological evidence for SR affecting depressive severity in the MDD patients and proposed potential biomarkers to identify the SR‐based risk phenotype of MDD individuals.

## Graphical abstract

Inter‐network functional connectivity of the orbital part of the right superior frontal gyrus and left supplementary motor area mediated the relationship between self‐regulation and depression. Self‐regulation could impact depressive symptoms via regulating inter‐network functional connectivity of thalamic subnuclei. Thalamic functional subnetworks could predict the self‐regulation and depressive severity of depressed individuals.

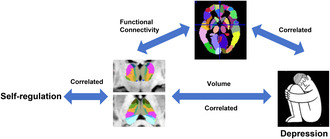

## 1 Introduction

Major depressive disorder (MDD), as a common and multifaceted mental disorder, affects around 185 million people across the world and leads to the continuously increasing mortality rate [1, 2]. However, the successful rate of treatment for MDD is extremely low. A critical obstacle is the high heterogeneity of depressive symptoms, which have differential neural basis and respond distinctively to treatments [3, 4, 5, 6].

Self‐regulation (SR) is a mental process involving emotional regulation, goal‐directed behavior, and cognitive control [7, 8]. The classical theoretical mode of SR is Goal System Theory [7], including Regulatory Focus Theory and Regulatory Mode Theory [9], delineating individual preferences to different methods in order to obtain goals. Individual SR differences could be manifested by distinct preferences for behaviors to obtain a goal, as promotion/locomotion‐oriented subjects tend to engage in behaviors in an optimistic manner, while prevention/assessment‐oriented subjects are prone to engage in conservative behaviors to avoid risk [10, 11]. SR is closely related to emotional regulation. Theoretically, subjects with distinct SR orientations feel different in the process of goal pursuit. For example, promotion/locomotion‐oriented subjects could enjoy more pleasure and satisfaction, while prevention/assessment‐oriented subjects experience more quiescence and composure [10, 11]. SR dysfunction was closely related to emotional symptoms [12, 13], and SR‐based interventions have been used for MDD patients in clinical practice as a result of significant effects on ameliorating depressive symptoms [14, 15, 16]. Specially, accumulating evidence has demonstrated that changes in SR strategies could predict adolescent depressive symptoms [17]. However, the neural basis linking SR with MDD remains unclear. Thus, delineating neural effects is pivotal to understanding the relationship between SR and MDD and beneficial to selecting therapeutic targets for depression.

Thalamus, consisting of multiple thalamic subnuclei (TS), relays information from and to cortex and subcortical structures, and is responsible for relaying sensory information, regulating consciousness, and cognitive control [18]. There is growing evidence of thalamic functional and structural role in MDD and emotional regulation [19, 20]. For example, functional activity of the thalamic mediodorsal nucleus (MD) increased in the acute phase of MDD [19], and thalamic activation played a vital role in emotional regulation development [21]. Crucially, specific TS relevant to depression is also involved in goal‐directed behavior, one of the core cognitive processes in SR [7, 8], by selecting sensory information [22], correcting action [23], value appraisal [24], and guiding action [25]. Thus, structural and functional roles of TS may be the neural basis of SR‐linking MDD. Notably, anatomical studies found multiple connections between TS and other structures but sparse connections among TS because interaction among TS was subjected to modulation of reticular nucleus [26, 27]. Besides, recent studies found that functional connectivity (FC) between specific thalamic subnuclei and other multiple structures, or thalamic functional subnetworks composed of a series of subnuclei and other structures, instead of a single thalamic subnucleus, was the real entity of thalamic functions [28, 29]. Therefore, exploring the FC of specific TS with other cortical and subcortical regions may be an effective method for revealing the functional role of TS.

In this study, we first segregated bilateral thalamus into 46 TS and parcellated the entire brain (except of cerebellum, brain stem, and bilateral thalamus) into 88 AAL brain regions and defined all 46 × 45/2 functional connectivity (FC) among 46 TS as thalamic intrinsic network and all 88 × 87/2 FC among 88 AAL regions as thalamic external network. Secondly, we investigated the volumetric difference of TS between healthy controls (HCs) and MDD patients and their significant correlation with SR subscales and depressive symptoms in MDD patients, as well as explored whether volumes of TS could mediate the relationship between SR subscales and depressive symptoms. Further, we studied the role of nodal FC within thalamic intrinsic or external networks and between the two networks in SR and MDD. Then, we mapped the functional mode of inter‐network FC of TS relevant to SR subscales and depressive symptoms via canonical correlation analysis (CCA). Finally, Network‐based Statistic‐Predict (NBS‐Predict), which combines Network‐based Statistic (NBS) with machine learning (ML) [30], was utilized to assess whether thalamic functional subnetworks could predict SR and depressive symptoms of MDD patients.

## 2 Materials and Methods

### 2.1 Participants and Study Approval

A total of 143 patients were recruited in the current study, including 68 HCs and 75 MDD patients. Two HC subjects were excluded due to excessive head motion during functional magnetic resonance imaging (fMRI) scanning and implausible thalamic segmentation. Therefore, we included 66 HCs and 75 MDD patients in further analysis. The detailed information for participants and study approval is described in Supplement.

### 2.2 Behavioral Measurements

All subjects underwent a comprehensive clinical assessment of their neurological and mental status, using HAMD‐24 for depression severity. We counted the seven subscales of the HAMD‐24, including anxiety, cognition, desperation, diurnal variation, retardation, sleep disorder, and loss of weight. The Regulatory Focus Questionnaire (RFQ), an 11‐item self‐report scale, contained subjective regulatory orientation of promotion and prevention. The Regulatory Mode Questionnaire (RMQ), a 24‐item self‐report scale, contains subjective regulatory orientation of locomotion and assessment. Predominance of RFQ, RFQ‐predominance, was obtained by subtracting prevention score from promotion score. Predominance of RMQ, RMQ‐predominance, was obtained by subtracting locomotion score from assessment score.

### 2.3 MRI Data Acquisition and Imaging Preprocess

Imaging was conducted on a Siemens 3.0 T scanner (Munich, Germany). The functional data were preprocessed using the DPABI toolbox in MATLAB 2013b. The detailed information for parameters of image data and image preprocessing is described in Supporting Information.

### 2.4 Construction of Thalamic Intrinsic and External Network

Thalamic intrinsic and external networks were constructed based on FC of 46 TS and 88 AAL brain regions. Then, intra‐network FC value of each AAL node by summing all FC within the thalamic external network; inter‐network FC of each thalamic node or AAL node by summing all inter‐network connectivity of this node. The detailed information for network construction and calculation of nodal intra‐ and inter‐network FC is described in Supplement.

### 2.5 Statistical Analysis

SPSS 26.0, RStudio 4.1.2, and MATLAB 2021b were used for statistical analysis in this study. For age, education level, and behavioral data, the Kolmogorov–Smirnov test was used to verify the normal distribution of parameters after *Z*‐score transformation. A two sample *t*‐test was utilized to compare continuous variables with normal distribution. A chi‐squared test was utilized to compare the group‐wise difference of gender. Furthermore, Pearson's correlation was used in the correlation analysis between SR factors and depressive symptoms after regressing out gender, age, and education. The significance level was set at *p* < 0.05. Bonferroni correction was used to avoid false‐positive rates in comparisons of behavioral data.

The Kolmogorov–Smirnov test was used to test the Gaussian distribution of imaging data after regressing of covariates. Group‐level difference of imaging data was performed by a two‐sample *t*‐test, and false discovery rate (Benjamini–Hochberg) was used for multiple comparisons at *q* < 0.05. The correlation between imaging data and behavioral data was performed by Pearson's correlation. For statistical analysis of volumetric data, gender, age, education, and volume of the ipsilateral whole thalamus (WT) were regressed out for TS, while gender, age, education, and the intracranial volume (ICV) instead of ipsilateral WT volume were regressed out for the whole left or right thalamus. For statistical analysis of functional data, gender, age, and education was regressed out. Exploratory mediation analysis was performed at the significant level of *p* < 0.05 with 5000 iterations of nonparametric bootstrapping. The CCA was used to explore the maximal correlation between the inter‐network FC of TS and subscales of behavioral data in order to avoid multicollinearity. In the first CCA, the network variate, HAMD variate, and SR variate were identified. In the second CCA, univariate correlation of network variate with HAMD variate and SR variate was analyzed in all samples and MDD subgroup. The threshold for statistical significance of CCA was set at *p* < 0.05.

The NBS‐Predict toolkit was utilized to assess whether thalamic subnetwork could predict individual SR and depressive symptoms [30]. NBS‐predict combines ML with NBS into a 5‐fold cross‐validation structure to perform connectome‐based prediction. Three kinds of ML were embedded, including Logistic Regression, Linear Support Vector Classification, and Linear Discriminant Analysis. The cross‐validation structure is repeated 10 times with 500 iterations. The edges with *p* < 0.05 were selected as initial connectivity for breath‐first‐search algorithm [31], which identified the connected components with suprathreshold edges. Subsequently, suprathreshold edges in the largest connected components were used to train predictive models for behavioral data through MLs. Finally, the optimal model, namely with the highest correlation with behavioral data, of three MLs was reported in this study.

## 3 Results

### 3.1 Demographic and Behavioral Data

Demographic and clinical characteristics of all participants are listed in Table 1. No significant difference was found in gender between HC and MDD, while age and education level were not matched. Compared with HC subjects, MDD patients showed higher scores of HAMD‐24 total and subscales and RMQ‐predominance, as well as lower scores of RFQ‐predominance, RFQ‐promotion, and RMQ‐locomotion. No significant difference was found in RFQ‐prevention or RMQ‐assessment between the two groups. All comparisons of behavioral data survived after Bonferroni correction (*p* < 0.05/14 = 0.0036).

**TABLE 1:** Demographic information and clinical characteristics.

	HC (*n* = 66)	MDD (*n* = 75)	*p*
Age, mean (SD), year	36.45 (12.04)	40.71 (11.36)	0.031
Gender (M/F)	30/36	33/42	0.867*
Education, mean (SD), year	12.92 (3.60)	10.57 (3.39)	< 0.001
HAMD‐24, mean (SD)	−0.90 (0.20)	0.79 (0.71)	< 0.001
HAMD‐anxiety, mean (SD)	−0.82 (0.21)	0.72 (0.85)	< 0.001
HAMD‐cognition, mean (SD)	−0.76 (0.21)	0.66 (0.95)	< 0.001
HAMD‐desperation, mean (SD)	−0.80 (0.22)	0.70 (0.88)	< 0.001
HAMD‐DV, mean (SD)	−0.53 (0.26)	0.47 (1.17)	< 0.001
HAMD‐retardation, mean (SD)	−0.80 (0.24)	0.71 (0.87)	< 0.001
HAMD‐sleep, mean (SD)	−0.64 (0.39)	0.56 (1.04)	< 0.001
HAMD‐weight, mean (SD)	0	0.27 (1.31)	< 0.001
RFQ‐predominance, mean (SD)	0.26 (0.96)	−0.23 (0.99)	0.003
RFQ‐prevention, mean (SD)	0.02 (1.00)	−0.02 (1.00)	0.846
RFQ‐promotion, mean (SD)	0.41 (0.96)	−0.36 (0.89)	< 0.001
RMQ‐predominance, mean (SD)	−0.31 (0.78)	0.27 (1.10)	0.001
RMQ‐locomotion, mean (SD)	0.29 (0.77)	−0.26 (1.11)	0.001
RMQ‐assessment, mean (SD)	−0.10 (0.86)	0.09 (1.10)	0.245
Total disease duration, mean (SD), month	N.A.	84.75 (97.40)	N.A.
Current disease duration, mean (SD), month	N.A.	4.73 (5.55)	N.A.
Suicide ideation (yes/no)	N.A.	50/25	N.A.
Suicide behavior (yes/no)	N.A.	4/71	N.A.
Family history (with/without)	N.A.	23/52	N.A.

*Note:* Unless indicated, data are presented as mean (standard deviation). N.A., data were not available. All abbreviations can be found in Table S1.

* *p* value was obtained by chi‐squared test. Other *p* values were obtained by two‐sample *t*‐test between HC and MDD.

The behavioral correlation between SR and depressive symptoms in the MDD subgroup is presented in Figure S1. For MDD subjects, RFQ‐promotion was related to HAMD‐24 (*r* = −0.30, *p* = 0.0083), HAMD‐cognition (*r* = −0.27, *p* = 0.0195), HAMD‐desperation (*r* = −0.35, *p* = 0.0020), and HAMD‐retardation (*r* = −0.26, *p* = 0.0227); RMQ‐predominance was related to HAMD‐cognition (*r* = 0.23, *p* = 0.0499) and HAMD‐desperation (*r* = 0.38, *p* = 0.0007); RMQ‐locomotion was related to HAMD‐24 (*r* = −0.25, *p* = 0.0320), HAMD‐desperation (*r* = −0.40, *p* = 0.0004) and HAMD‐retardation (*r* = −0.23, *p* = 0.0465).

### 3.2 Volumetric Alterations of TS

**FIGURE 1:**
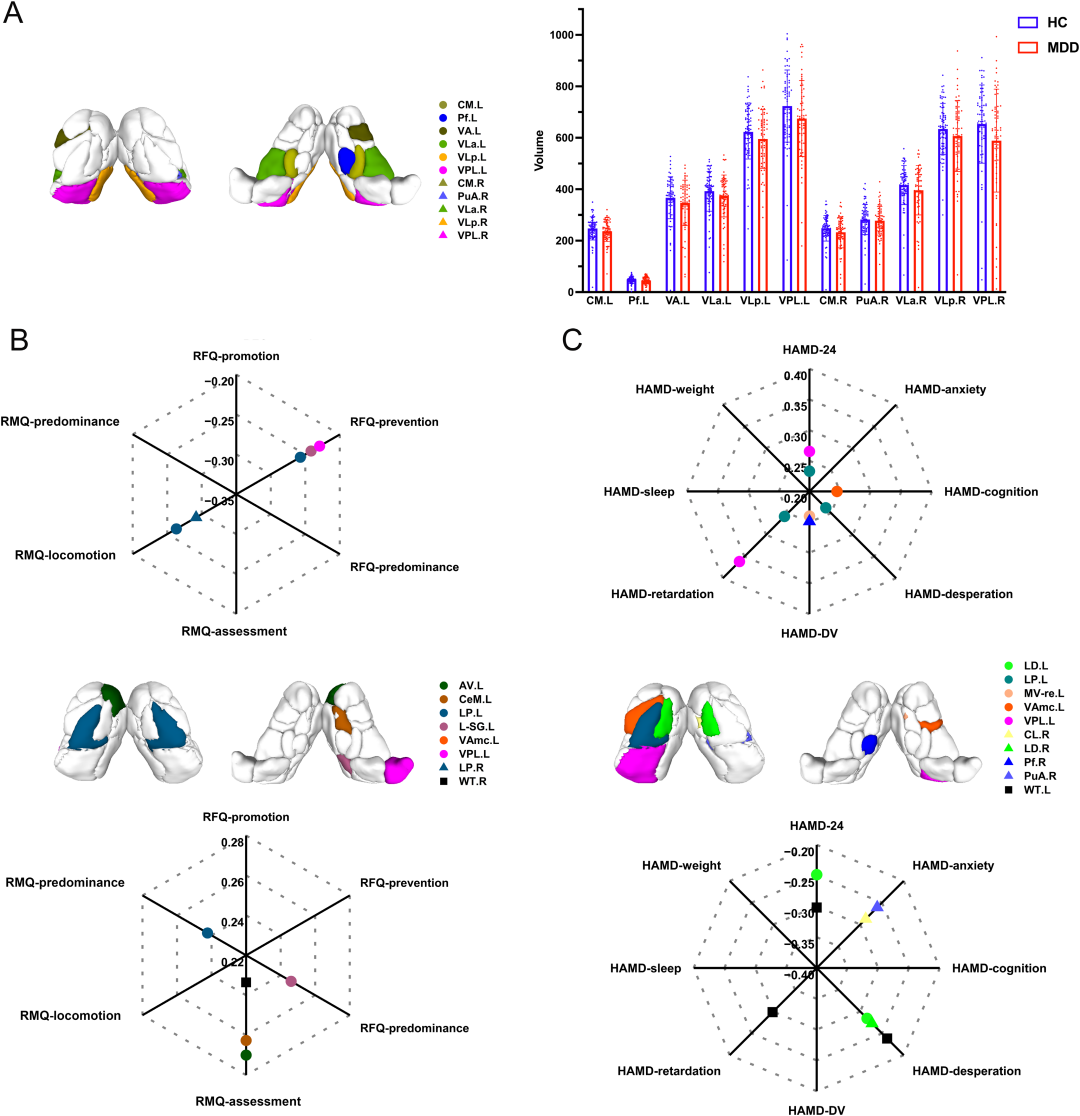
Group‐level comparison and behavioral correlation of thalamus volumes. (A) Map and comparison of thalamic subnuclei with significantly different volumes between HC and MDD, with every dot in the bar plot representing an individual volumetric parameter. (B and C) Map of thalamic subnuclei and the left entire thalamus with volumes related to SR (left) and depressive symptoms (right), with dots in the radar plot showing the correlation coefficients. Circles represent regions in the left hemisphere, whereas triangles represent regions in the right hemisphere. All abbreviations can be found in Table S1.

Figure 1 illustrates group‐level volumetric comparison for TS and the volumetric association of TS with SR and depressive symptoms in MDD patients. Eleven TS showed decreased volumes in MDD patients, including CM.L, Pf.L, VA.L, VLa.L, VLp.L, and VPL.L in the left thalamus and CM.R, PuA.R, VLa.R, VLp.R, and VPL.R in the right thalamus, but no findings survived after Bonferroni correction for multiple comparisons. Besides, no significant group‐level difference was found in the volumes of the entire left or right thalamus. The detailed information for the abbreviation of the TS was described in Table S1.

Volumetric changes of several TS were related to SR or depressive symptoms, respectively, while no significantly mediated effect was found through mediation analysis. Specially, volumetric changes of TS related to SR were predominantly in the left thalamus, including AV.L, CeM.L, LP.L, L‐SG.L, VAmc.L and VPL.L, while volumetric change of the right whole thalamus was related to RMQ‐assessment. For depressive symptoms, volumes of TS at the left side showed mainly positive correlation with HAMD‐24 total and subscale scores. For example, VPL.L was positively related to HAMD‐24 and HAMD‐retardation, and LD.L was positively related to HAMD‐24, HAMD‐desperation, and HAMD‐retardation. However, volume of the entire left thalamus was negatively related to HAMD‐24, HAMD‐desperation, and HAMD‐retardation. The detailed information was described in Figure 1.

### 3.3 Alterations of Nodal FCs Within Thalamic Intrinsic, External Network, and Between Networks

**FIGURE 2:**
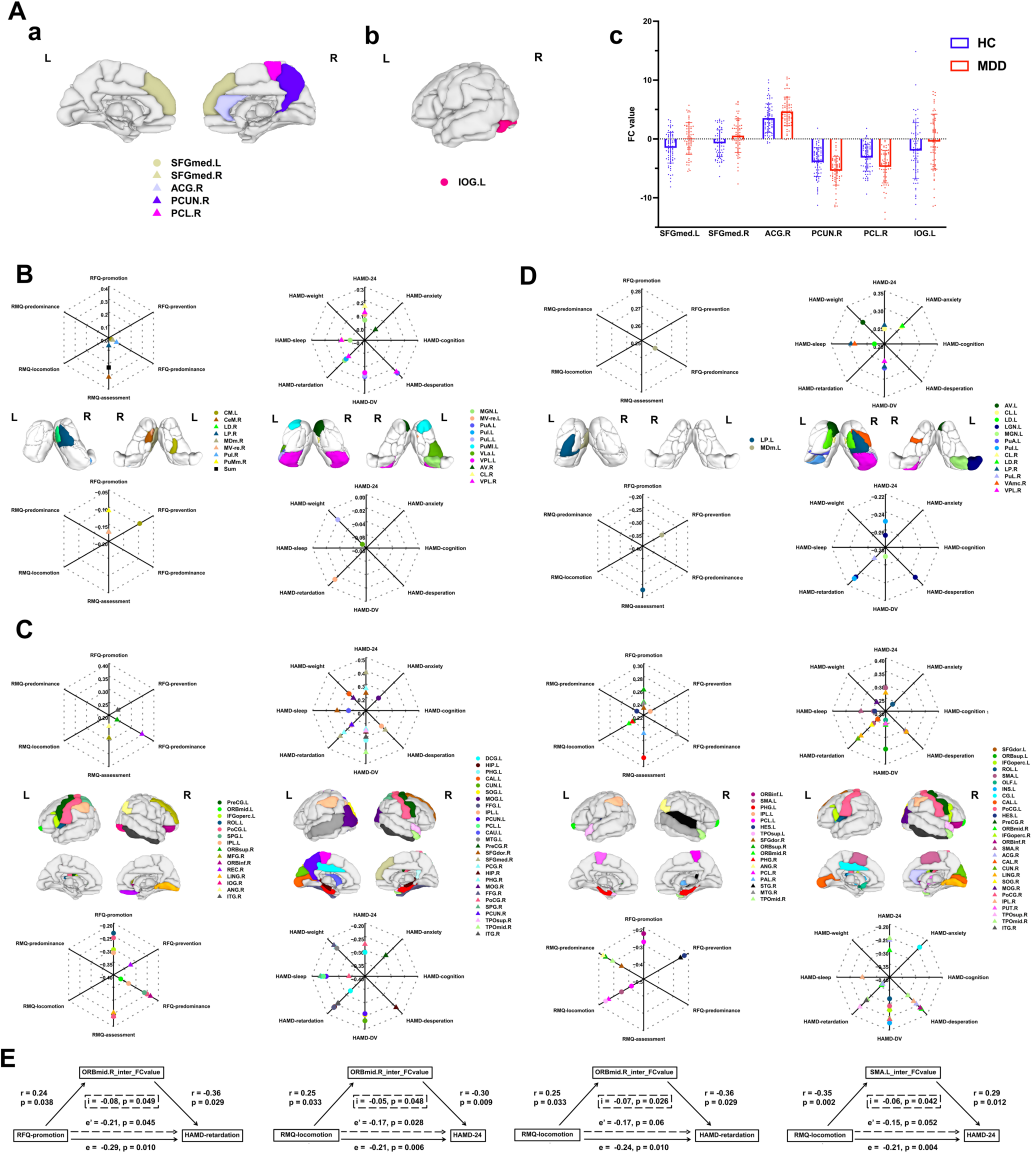
Group‐level comparison and behavioral correlation of nodal intra‐ and inter‐network functional activity. (A) Map of nodes with significant different intra‐network FC (a) and inter‐network FC (b) between HC and MDD, and comparison of nodal FC value (c), with every dot in the bar plot representing individual parameter of FC value and the black dotted line indicating where the FC value equals 0. (B) Map of thalamic subnuclei with intra‐network FC related to SR (left) and depressive symptoms (right). (C) Map of AAL regions with intra‐network FC related to SR (left) and depressive symptoms (right). (D) Map of nodes with inter‐network FC related to SR (left) and depressive symptoms (right). (E) Inter‐network FCs of ORBmid.R and SMA.L mediate the correlation between SR and depressive symptoms. Radar plots show the correlation coefficients of nodal FC with the total score or subscales of SR or HAMD‐24. Circles represent regions in the left hemisphere, whereas triangles represent regions in the right hemisphere. *e*, total effect of mediation analysis; *e*′, indirect effect of mediation analysis; *i*, mediated effect of mediation analysis; *p*, *p* values of *r*; *r*, correlation coefficient. All abbreviations can be found in Table S1.

Group‐level difference of nodal FC and association of those with RFQ, RMQ, and HAMD‐24 total and subscales were shown in Figure 2. Specifically, no significant group‐level difference of TS FC within the intrinsic network was observed. Within the external network, MDD patients showed higher FC at SFGmed.L, SFGmed.R, and ACG.R and lower FC at PCUN.R and PCL.R than those of HC subjects. For comparison of nodal FC between the two networks, IOG.L was the only node that showed a stronger connection to the intrinsic network, while nodes of TS showed no significant changes in functional connections to the external network after FDR correction at *q* < 0.05 (*p* < 0.012).

Nodal FC of TS related to SR in MDD patients were mainly located at right TS, including CeM.R, LD.R, LP.R, MDm.R, MV‐re.R, PuI.R, and PuMm.R, as FC values within the intrinsic network of left TS and between networks were scarcely related to SR. Of note, the sum of nodal FC within the intrinsic networks was significantly related to RMQ‐assessment. On the contrary, correlation between SR and FC of AAL regions observed that FC of ORBmid.L, IPL.L, and ANG.R were significantly related to SR both within the external network and between networks. Symptom‐specific correlated pattern of TS FC found that MGN.L and VPL.R showed symptom‐related FC both within the intrinsic network and between networks. Meanwhile, the correlated map of FC of AAL regions indicated that both intra‐network and inter‐network FC of 6 AAL nodes, including DCG.L, CAL.L, PreCG.R, PoCG.R, MOG.R, and TPOmid.R, were associated with MDD, though at different dimensions of symptoms. Specifically, both intra‐ and inter‐network FC of CL.L were positively related to HAMD‐24 total score. Moreover, both intra‐ and inter‐network FC of MOG.R were positively related to HAMD‐weight. The detailed information was described in Figure 2B–D.

Mediation analysis further revealed that inter‐network FC of ORBmid.R and SMA.L, namely nodal FC with thalamic intrinsic network, were involved in the mediation of the relationship between SR and depressive symptoms. Inter‐network FC of ORBmid.R could partially mediate the association of RFQ‐promotion with HAMD‐retardation and the association of RMQ‐locomotion with HAMD‐24. Whereas inter‐network FC of ORBmid.R was a full mediator between RMQ‐locomotion and HAMD‐retardation. Besides, inter‐network FC of SMA.L could fully mediate the association between RMQ‐locomotion and HAMD‐24. The detailed information was described in Figure 2E.

### 3.4 Canonical Correlation of Inter‐Network Functional Activity of TS With SR and Depressive Symptoms

**FIGURE 3:**
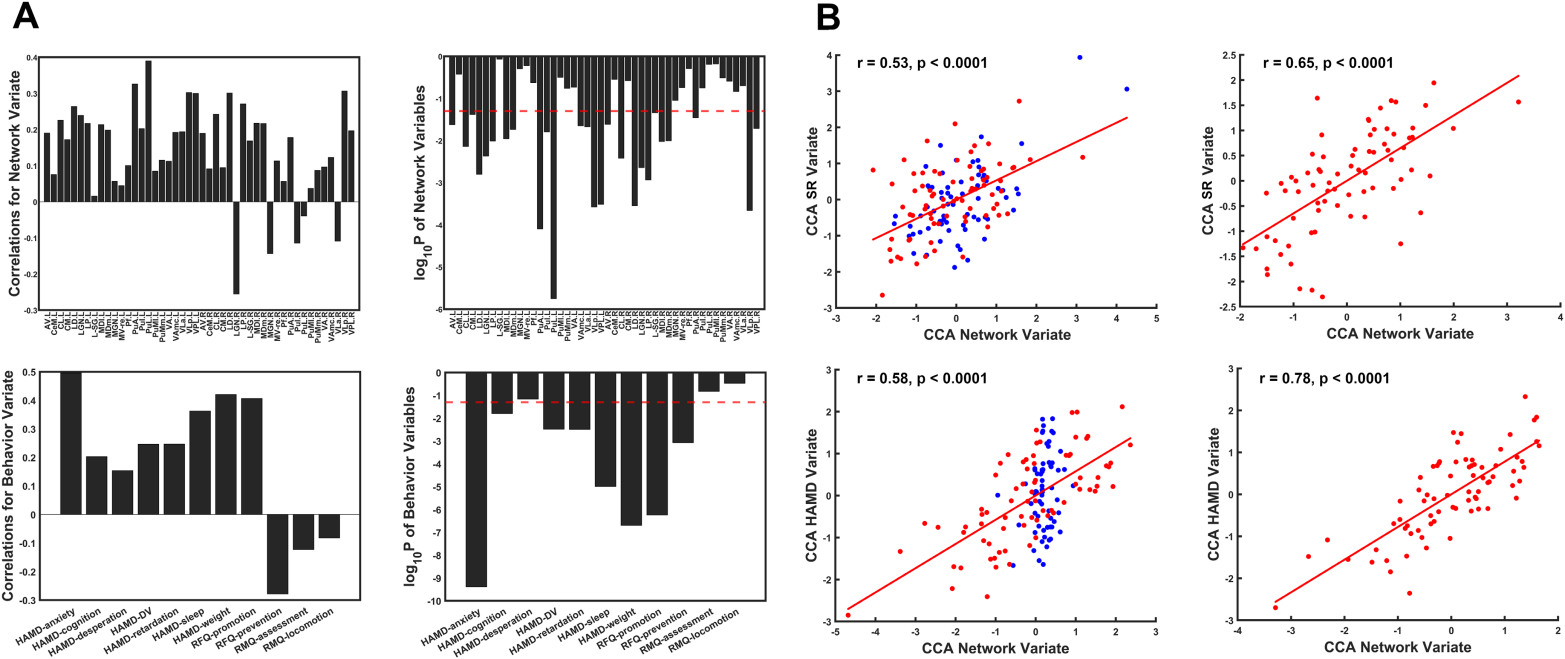
CCA mode among thalamic inter‐network variables, SR, and depressive symptoms. (A) The CCA mode of 46 thalamic inter‐network FC values, seven HAMD subscales, and four SR subscales. Red dotted lines indicate *p* = 0.05. (B) Correlation between network variate and SR variate or HAMD variate in all subjects (left) and MDD (right), with blue dots representing variates of HCs and red dots representing variates of MDD patients. All abbreviations can be found in Table S1.

The results obtained from the exploratory canonical correlation are presented in Figure 3. The CCA pattern was based on 46 network variables, seven HAMD variables, two RFQ variables, and two RMQ variables. By first CCA, inter‐network FC of 25 TS bilaterally (including AV.L, CL.L, CM.L, LD.L, LGN.L, LP.L, MDl.L, MDm.L, PuA.L, Pul.L, PuL.L, VAmc.L, VLa.L, VLp.L, VPL.L, AV.R, CL.R, LD.R, LGN.R, LP.R, L‐SG.R, MDl.R, MDm.R, PuA.R, VLp.R, VPL.R) was canonically correlated with all HAMD subscales, except of HAMD‐desperation, and two RFQ subscales (*r* = 0.7999, *p* < 0.0001). Specially, except for LGN.R, all correlated TS were positively related to the network variate. In terms of behavioral variables, RFQ‐prevention was the only variable which accounted for the negative correlation with the behavioral variate. In the correlation analysis of CCA variates, the network variate was positively related to SR variate (*r* = 0.53, *p* < 0.0001) and HAMD variate (*r* = 0.58, *p* < 0.0001) in all subjects. Moreover, MDD subgroups presented enhanced correlations of network variate with SR variate (*r* = 0.65, *p* < 0.0001) and depressive variate (*r* = 0.78, *p* < 0.0001).

### 3.5 Thalamic Subnetworks Predict SR and Depressive Symptoms in MDD Patients

**FIGURE 4:**
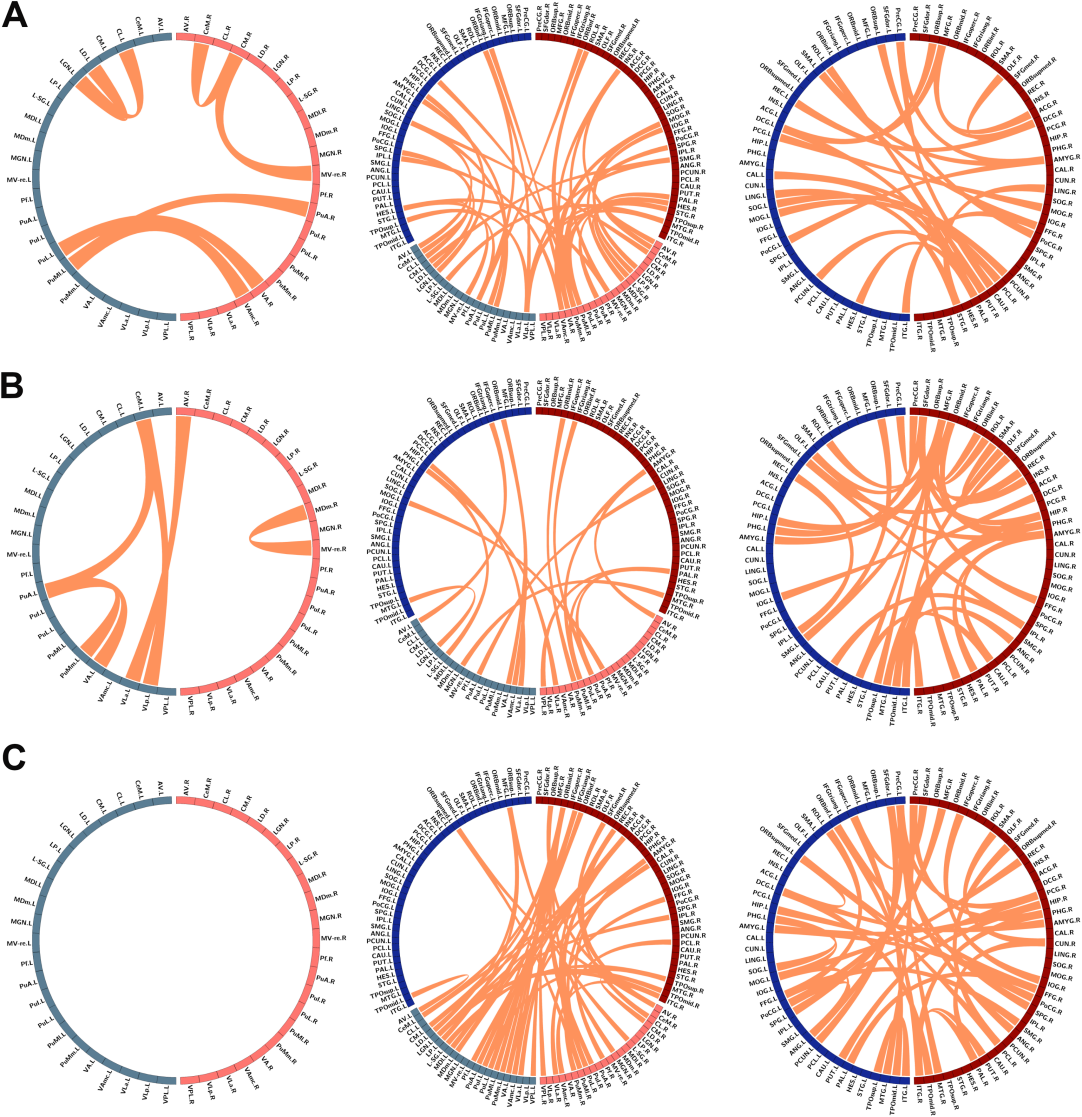
Thalamic functional subnetworks predict SR and depressive symptoms. (A) Pattern of thalamic functional subnetwork to predict RFQ‐prevention (*r* = 0.24, *p* = 0.039). (B) Pattern of the thalamic functional subnetwork to predict RMQ‐assessment (*r* = 0.21, *p* = 0.015). (C) Pattern of the thalamic functional subnetwork to predict HAMD‐retardation (*r* = 0.28, *p* = 0.049). The three circles represent FCs within thalamic subnuclei (left), between thalamic subnuclei and AAL regions (middle), and within AAL regions (right). All abbreviations can be found in Table S1.

Three thalamic functional subnetworks, which can predict the level of RFQ‐prevention, RMQ‐assessment, and HAMD‐retardation of MDD individuals, were shown in Figure 4. Thalamic connectivity between right and left thalamus, including PuL.L‐VAmc.R, PuMl.L‐VA.R, and PuMl.L‐PuA.R, was closely related to the RFQ‐prevention subnetwork. Thalamic connectivity within the left thalamus played a critical role in RMQ‐assessment subnetwork. Whereas, thalamic intrinsic connectivity showed no association with the HAMD‐retardation subnetwork at the weighted threshold of 1.0. Besides, the predictive ability of the HAMD‐retardation subnetwork depended largely on the connectivity of TS to other brain regions, and connectivity within cortical regions was associated with all three subnetworks. Indicating that FC changes of thalamus, instead of volumetrics, represent a more vulnerable response to SR or depression.

## 4 Discussion

This study found that volumes of TS and FCs related to thalamic networks were correlated with SR subscale and depressive symptoms. Specially, inter‐network FC of ORBmid.R and SMA.L could mediate the relationship between SR and MDD. Furthermore, based on FCs of TS to all nodes of the external network, this study analyzed the inter‐network functional mode of 25 TS and could regulate the relationship between SR and MDD. Crucially, functional networks associated with TS could predict SR and depressive symptoms for MDD patients.

First, we found no group‐wise volumetric difference for TS or the entire bilateral thalamus after FDR correction. Previous results on volumetric comparison of TS were inconsistent [32, 33], which might result from diverse segmentation methods and sample sizes. In volumetric correlation analysis, we found AV.L volume was positively correlated to RMQ assessment. This finding was in accordance with preferential evaluation relying on the learned value of MDD patients who are oriented to assessment and hinted memory function of this subnuclei during the information process [10, 34]. Furthermore, the opposite correlation of VPL.L with both HAMD‐retardation and RFQ‐prevention indicated the severity of individual psychomotor retardation might be due to individual excessive concern for failure. Noteworthily, we also provided imaging evidence in depressed patients for the role of VAmc‐PFC circuits in coordinating cognitive functions of cortical regions [35, 36, 37], the role of CeM in involving in cognitive process [38], and the role of Pf in regulating depression‐related discomforts on circadian rhythms in contexts of enduring depression. Previous studies found that inhibition of amygdala‐Pf‐secondary somatosensory cortex could lead to pain perception of depression‐like states [39], and Pf‐related circuits were responsible for correcting and shifting behaviors according to sensorimotor information in cognitive process [40]. Hence, we speculate that Pf atrophy leads to lasting negative proprioception, which aggravates depression, and can be a potential rhythmic mechanism to regulate depression.

Second, in the group‐wise comparison of nodal FC, increased intra‐network FC of SFGmed.L/R and ACG.R and decreased PCUN.R and PCL.R were parallel with the role of PFC function in emotional regulation as well as reduced antioxidant and neurotransmitters in neuropathology of MDD [41, 42, 43]. Inter‐network FC alteration of IOG.L suggested the sensory and visual information process of thalamus played a role in the neuropathology of MDD. In addition, inter‐network FC of ORBmid.R and SMA.L could mediate the correlation of RFQ‐promotion and RMQ‐locomotion with HAMD‐24 and HAMD‐retardation. Orbital PFC was a pivotal region to thalamocortical circuits related to MD and regulated operation flexibility [44, 45]. SMA was a recent finding related to psychomotor retardation. Repetitive transcranial magnetic stimulation over SMA has been validated to reduce psychomotor retardation in MDD [46]. Moreover, altered FC between SMA and thalamus was also found in depressed patients with retardation [47]. Beyond prior results, our findings further emphasized the pathological role of SR, namely decreased individual willingness to take promotional measures and act immediately to attain goals, in the relationship between ORBmid.R/SMA.L‐thalamic loops and psychomotor retardation of depression. Furthermore, we found both intra‐ and inter‐network FC of CL.L were related to severity of depression. At the same time, both intra‐ and inter‐network FC of MOG.L were related to weight change. Previous studies had recognized functions of cortico‐striatal‐CL loops in motivational behaviors and consciousness [48, 49], occipital cortex in weight change and emotional regulation [50, 51, 52], and MOG‐CL connectivity in motivational behavior via regulating arousal level and visuospatial attention [53]. Thus, based on the above findings, we postulated that MOG‐CL circuits may constitute neural substrates of the relationship between SR and weight change related to depression.

Third, through CCA, we identified the inter‐network FC mode of TS, which was subjected to individual levels of promotion and prevention orientation and depressive severity. Specially, the correlation of network variate with SR variate and HAMD variate increased from all subjects to MDD patients. A majority of TS in this CCA mode have been discerned in motivational behaviors. For instance, AV and LD both interconnected with retrosplenial cortex and regulated mnemonic function [34, 54]. As well, CL played a role in memory, attention, and planning [55], while CM could inhibit reward‐response to facilitate behavior shifting [56]. Additionally, LGN was assisted in vigilance transition and behavioral guidance under the light stimuli [57]. Importantly, MD interacted with PFC to change behavioral strategy [58, 59, 60, 61]. Specially, MDm was mainly responsible for memory acquisition and referring to learning and evaluating previous experience [41, 62, 63], while MDl mainly focused on guidance of motivational behaviors [64]. Interestingly, the coordinating subnuclei VAmc, which facilitated communication among PFC, parietal cortex, and temporal cortex [35, 36], also engaged in this mode, suggesting that thalamus was also of vital importance to information without the need of thalamic relay and process. Besides, several cortico‐thalamic circuits involving emotional regulation and antidepressant effects were related to this CCA mode, including LGN‐habenula [57], and MDm‐amygdala [65]. Furthermore, this mode was correlated positively with promotion level and negatively with prevention level, suggesting enhanced RFQ‐promotion and weakened RFQ‐prevention could ameliorate the severity of depressive symptoms. Thus, this inter‐network FC mode of TS reflected how the two behaviors are linked in MDD patients and emphasized several neurological functions, including memory encoding, sensory processing, behavioral correction and guidance, and decision‐making in this relationship.

Finally, we identified three thalamic functional subnetworks that were correlated with RFQ‐prevention, RMQ‐assessment, and HAMDretardation. Interactions between TS and PFC regions were extensively identified among the three significant subnetworks, which provided imaging evidence for the role of cortical‐striatal‐thalamic circuits in affective disorders and goal‐directed behaviors [37, 66]. Besides, each subnetwork represented special characteristics, which was consistent with the view that entities of thalamic functions were subnetworks of TS [67]. For the RFQ‐prevention subnetwork, intra‐ and inter‐network FC of intralaminar nuclei at consciousness‐related parietal‐striatal‐thalamic circuit was positively associated with RFQ‐prevention subscale [48, 49], suggesting compromised sensory processing and perceptual experience in MDD subjects. For RMQ‐assessment subnetwork, intense involvement of the pallidum‐thalamic‐amygdala‐hippocampal‐PFC loop highlighted memory functions of cortico‐thalamic circuitry in emotional regulation [41]. For the HAMD‐retardation subnetwork, intense correlation of various TS and prefrontal cortex, amygdala, and temporary lobes suggested the emotional and cognitive role of the right cortico‐thalamic circuit in psychomotor retardation of MDD.

Several limitations of this study need to be considered. First, the sample size is comparably small so that further validation in a larger sample from multiple sites is in great demand to generalize our results in the population. Second, this is a cross‐sectional study, which limits the research into the role of TS in the relationship between SR and depressive symptoms at different stages of MDD. Third, other SR factors, measuring SR function from other perspectives instead of positive and negative regulation orientation, may implicate depressive severity and need investigation in the future.

## 5 Conclusion

In conclusion, this study found that volumes and FCs relevant to TS were related to SR and depressive symptoms in MDD patients. Specially, inter‐network FC of ORBmid.R and SMA.L could mediate the relationship between SR subscale and depressive symptoms, which provided directions for further studies on the role of thalamic circuits in goal‐directed behavior and depression. Furthermore, RFQ‐promotion and RFQ‐prevention could have an influence on depressive symptoms via regulating inter‐network FC of TS. Crucially, thalamic functional subnetworks could be potential biomarkers of RFQprevention, RMQ‐assessment, and HAMD‐retardation for MDD individuals. Thus, these findings provided neurological evidence for the correlation of SR with MDD and provided potential biomarkers for identifying the SR‐based risk phenotype of MDD.

## Author Contributions

**Zhang Ling:** conceptualization, data analysis, writing the original draft, and visualization. **He Cancan, Liu Xinyi**, and **Fan Dandan:** data collection. **Zhang Haisan** and **Zhang Hongxing:** funding acquisition. **Xie Chunming:** conceptualization, modification and proofreading, funding acquisition, and project administration.

## Conflicts of Interest

The authors declare no conflicts of interest.

## Supporting information


Data S1.

## Data Availability

The data that support the findings of this study are available on request from the corresponding author. The data are not publicly available due to privacy or ethical restrictions.
