# Supplementary material for: Thalamic Volumes and Functional Networks Linked With Self‐Regulation Dysfunction in Major Depressive Disorder
Source: CNS Neurosci Ther. 2024 Nov 10;30(11):e70116. doi: 10.1111/cns.70116 (PMC11551040; doi:10.1111/cns.70116)
Supplement: Supplementary file 1 — Data S1. [file CNS-30-e70116-s001.docx]

**Supplemental Material**

**Title: Thalamic Volumes and Functional Networks linked with Self-regulation Dysfunction in Major Depressive Disorder**

**This file includes:**

Methods

Supplementary Table S1

Supplementary Figure S1

1. **Methods**

**1.1. Participants and study approval**

A total of 143 patients were recruited in the current study, including 68 HCs and 75 MDD patients. Power Analysis and Sample Size (Hintze, J. NCSS, LLC. Kaysville, Utah, USA. www.ncss.com) was used to calculate the sample power for the clinical study. All subjects were Chinese Han population and right-handed. MDD patients were recruited through inpatients at the Department of Psychiatry in Henan Provincial Mental Hospital. HCs were recruited through local community posting and media advertising. Inclusion criteria for MDD patients: (1) meeting the diagnostic criteria for MDD using a Structured Clinical Interview by two experienced neuropsychiatrists (Chunming Xie and Hongxing Zhang) according to the Diagnostic Statistical Manual of Mental Disorder, Fifth Edition (DSM-V). (2) the score ≥ 8 which was measured by 24-item Hamilton Depression Rating Scale (HAMD-24). (3) drug naïve or drug free for longer than three weeks. (4) age between 18 and 55. Inclusion criteria for HCs: HAMD-24 score ≤ 7. Exclusion criteria for all subjects: (1) other major psychiatric disorders or neurodegenerative disease history. (2) substance abuse, head trauma, or loss of consciousness. (3) contraindications to MRI scanning. The ethics committee of Henan Provincial Mental Hospital Affiliated with Xinxiang Medical University approved this research protocol (approval ID:2017-08), and all participants or their legally authorized representatives signed the written informed consents for current study. Two HC subjects were excluded due to excessive head motion during functional magnetic resonance imaging (fMRI) scanning and implausible thalamic segmentation. Therefore, we included 66 HCs and 75 MDD patients in further analysis.

**1.2. Behavioral measurements**

We counted the seven subscales of the HAMD-24 including anxiety, cognition, desperation, diurnal variation, retardation, sleep disorder and loss of weight. The Regulatory Focus Questionnaire (RFQ), an 11-item self-report scale, contained subjective regulatory orientation of promotion and prevention. The Regulatory Mode Questionnaire (RMQ), a 24-item self-report scale, contained subjective regulatory orientation of locomotion and assessment. Predominance of RFQ, RFQ-predominance, was obtained by subtracting prevention score from promotion score. Predominance of RMQ, RMQ-predominance, was obtained by subtracting locomotion score from assessment score.

**1.3. MRI data acquisition**

Imaging was conducted on Siemens 3.0T scanner (Munich, Germany). T1-weighted anatomical scans, resting-state functional scans of approximately 8 min and routine axial T2-weighted scans were acquired from all participants. All subjects were instructed to relax, maintain closed eyes, and stay awake during scans, with earplugs to reduce noise and stabilizers to immobilize subjects.The parameters for T1 data were repetition time = 1900 ms, echo time = 2.48 ms, flip angle = 90°, acquisition matrix = 256 × 256, field of view = 240 × 240 mm, thickness = 1.0 mm, gap = 0 mm, number of slices = 176, and number of excitations = 1.0. The parameters for functional data were repetition time = 2000 ms, echo time = 25 ms, flip angle = 90°, acquisition matrix = 64 × 64, field of view = 240 × 240 mm, thickness = 4.0 mm, gap = 0 mm, number of excitations = 1.0, and number of slices = 36. The sequence parameters of the Diffusion tensor imaging (DTI) data were repetition time = 10000 ms, echo time = 90 ms, flip angle = 90°, field of view = 256 × 256 mm^2^, acquisition matrix = 128 × 128, 30 diffused weighted directions b = 1000 s/mm^2^, 1 non-diffused weighted direction b0 = 0, thickness = 2.0 mm, gap = 0 mm, number of slices = 70.

**1.4. Imaging preprocess**

**1.4.1. Thalamic segmentation**

T1 images were automatically processed via command “recon-all”. The processing pipeline included motion correction, signal intensity normalization, automated Talairach transformation, removal of nonbrain tissue, automated topology correction. DTI images were automatically processed by running TRACULA via command “trac-all”, which includes FSL’s eddy current and subject motion correction. Subsequently, bilateral thalamus was segmented into 46 subnuclei in total via shell script “segmentThalamicNuclei_DTI.sh” by joint segmentation from T1 and DTI^31, 32^. Finally, all thalamic segmentations were inspected visually and a segmentation which showed unmatched thalamic boundaries to that in T1 image was excluded. All processes in thalamic segmentation were performed using FreeSurfer version 7.4.1.

**1.4.2. Functional MRI data preprocessing**

The functional data were preprocessed using the DPABI toolbox in MATLAB 2013b with following steps^33^: (1) discard the first 10 volume. (2) correct for slice timing, realign, and spatially normalize to the standard Montreal Neurological Institute space using the DARTEL toolbox. (3) smooth with a 6-mm full-width half-maximum kernel. (4) remove confounding factors, including: 24 motion parameter, cerebrospinal fluid signals, white matter signals, the global mean signal, and an overall linear trend. (5) inspect excessive motion thresholds (translational movement >2.5 mm or rotational movement >3°) and mean framewise displacement (> 0.2 mm). (6) apply a bandpass filter between 0.01 and 0.08 Hz. Subsequently, all volumes were resampled into 1 × 1 × 1 mm^3^ via trilinear interpolation, using NIFTI_20140122 package embedded in MATLAB 2013b. All data in this study conformed above quality control metric.

**1.4.3. Construction of thalamic intrinsic and external network**

For nodes of thalamic subnuclei, we realign individual thalamic atlas of 46 subnuclei into MNI152 space and register it to individual fMRI image using affine and rigid body transformation. For AAL nodes, we used AAL90 atlas to partition the brain into 88 cortical and subcortical regions (except of bilateral thalamus). Pearson’s correlation was used to estimate the FC between all pairs of regions of interest areas in all subjects. We construct thalamic intrinsic and external network based on FC of the all nodes. The FC of each thalamic subnuclei with all 88 AAL regions was reckoned as thalamic inter-network connectivity, and that of each AAL node with all 46 thalamic node was reckoned as AAL inter-network connectivity. Subsequently, Fisher z-transformation was applied for all FC. Then, intra-network FC value of each thalamic node was calculated by summing all FC within thalamic intrinsic network; Intra-network FC value of each AAL node was calculated by summing all FC within thalamic external network; Inter-network FC of each thalamic node or AAL node was calculated by summing all inter-network connectivity of this node.

**Table S1. List of abbreviations used in Figures and Tables.**

| **Abbreviation** | **Full Name** |
| --- | --- |
| HAMD-24 | 24-item Hamilton Depression Scale |
| HAMD-anxiety | Hamilton Depression Scale-anxiety factor |
| HAMD-cognition | Hamilton Depression Scale-cognitive disturbance factor |
| HAMD-desperation | Hamilton Depression Scale-desperation factor |
| HAMD-DV | Hamilton Depression Scale-diurnal variation factor |
| HAMD-retardation | Hamilton Depression-retardation factor |
| HAMD-sleep | Hamilton Depression Scale-sleep disorder factor |
| HAMD-weight | Hamilton Depression Scale-loss of weight factor |
| RFQ | Regulatory Focus Questionnaire |
| RFQ-predominance | Regulatory Focus Questionnaire-regulatory focus predominance |
| RFQ-prevention | Regulatory Focus Questionnaire-prevention focus |
| RFQ-promotion | Regulatory Focus Questionnaire-promotion focus |
| RM-predominance | Regulatory Mode-regulatory mode predominance |
| RM-locomotion | Regulatory Mode-locomotion |
| RM-assessment | Regulatory Mode-assessment |
| PreCG.L | Left precentral gyrus |
| SFGdor.L | Left superior frontal gyrus, dorsolateral |
| ORBsup.L | Left superior frontal gyrus, orbital part |
| MFG.L | Left middle frontal gyrus |
| ORBmid.L | Left superior frontal gyrus, orbital part |
| IFGoperc.L | Left inferior frontal gyrus, opercular part |
| IFGtriang.L | Left inferior frontal gyrus, triangular part |
| ORBinf.L | Left inferior frontal gyrus, orbital part |
| ROL.L | Left Rolandic operculum |
| SMA.L | Left supplementary motor area |
| OLF.L | Left olfactory cortex |
| SFGmed.L | Left superior frontal gyrus, medial |
| ORBsupmed.L | Left superior frontal gyrus, medial orbital |
| REC.L | Left gyrus rectus |
| INS.L | Left insula |
| ACG.L | Left anterior cingulate and paracingulate gyri |
| DCG.L | Left median cingulate and paracingulate gyri |
| PCG.L | Left posterior cingulate and paracingulate gyri |
| HIP.L | Left hippocampus |
| PHG.L | Left parahippocampal gyrus |
| AMYG.L | Left amygdala |
| CAL.L | Left calcarine fissure and surrounding cortex |
| CUN.L | Left cuneus |
| LING.L | Left lingual gyrus |
| SOG.L | Left superior occipital gyrus |
| MOG.L | Left middle occipital gyrus |
| IOG.L | Left inferior occipital gyrus |
| FFG.L | Left fusiform gyrus |
| PoCG.L | Left postcentral gyrus |
| SPG.L | Left superior parietal gyrus |
| IPL.L | Left inferior parietal, but supramarginal and angular gyri |
| SMG.L | Left supramarginal gyrus |
| ANG.L | Left angular gyrus |
| PCUN.L | Left precuneus |
| PCL.L | Left paracentral lobule |
| CAU.L | Left caudate nucleus |
| PUT.L | Left lenticular nucleus, putamen |
| PAL.L | Left lenticular nucleus, pallidum |
| HES.L | Left heschl gyrus |
| STG.L | Left superior temporal gyrus |
| TPOsup.L | Left temporal pole: superior temporal gyrus |
| MTG.L | Left middle temporal gyrus |
| TPOmid.L | Left temporal pole: middle temporal gyrus |
| ITG.L | Left inferior temporal gyrus |
| PreCG.R | Right precentral gyrus |
| SFGdor.R | Right superior frontal gyrus, dorsolateral |
| ORBsup.R | Right superior frontal gyrus, orbital part |
| MFG.R | Right middle frontal gyrus |
| ORBmid.R | Right superior frontal gyrus, orbital part |
| IFGoperc.R | Right inferior frontal gyrus, opercular part |
| IFGtriang.R | Right inferior frontal gyrus, triangular part |
| ORBinf.R | Right inferior frontal gyrus, orbital part |
| ROL.R | Right Rolandic operculum |
| SMA.R | Right supplementary motor area |
| OLF.R | Right olfactory cortex |
| SFGmed.R | Right superior frontal gyrus, medial |
| ORBsupmed.R | Right superior frontal gyrus, medial orbital |
| REC.R | Right gyrus rectus |
| INS.R | Right insula |
| ACG.R | Right anterior cingulate and paracingulate gyri |
| DCG.R | Right median cingulate and paracingulate gyri |
| PCG.R | Right posterior cingulate and paracingulate gyri |
| HIP.R | Right hippocampus |
| PHG.R | Right parahippocampal gyrus |
| AMYG.R | Right amygdala |
| CAL.R | Right calcarine fissure and surrounding cortex |
| CUN.R | Right cuneus |
| LING.R | Right lingual gyrus |
| SOG.R | Right superior occipital gyrus |
| MOG.R | Right middle occipital gyrus |
| IOG.R | Right inferior occipital gyrus |
| FFG.R | Right fusiform gyrus |
| PoCG.R | Right postcentral gyrus |
| SPG.R | Right superior parietal gyrus |
| IPL.R | Right inferior parietal, but supramarginal and angular gyri |
| SMG.R | Right supramarginal gyrus |
| ANG.R | Right angular gyrus |
| PCUN.R | Right precuneus |
| PCL.R | Right paracentral lobule |
| CAU.R | Right caudate nucleus |
| PUT.R | Right lenticular nucleus, putamen |
| PAL.R | Right lenticular nucleus, pallidum |
| HES.R | Right heschl gyrus |
| STG.R | Right superior temporal gyrus |
| TPOsup.R | Right temporal pole: superior temporal gyrus |
| MTG.R | Right middle temporal gyrus |
| TPOmid.R | Right temporal pole: middle temporal gyrus |
| ITG.R | Right inferior temporal gyrus |
| AV.L | Left anteroventral nucleus |
| CeM.L | Left centromedian nucleus |
| CL.L | Left central lateral nucleus |
| CM.L | Left central medial nucleus |
| LD.L | Left laterodorsal nucleus |
| LGN.L | Left lateral geniculate nucleus |
| LP.L | Left lateral posterior nucleus |
| L-SG.L | Left limitans (suprageniculate) |
| MDl.L | Left mediodorsal lateral magnocellular nucleus |
| MDm.L | Left mediodorsal medial magnocellular nucleus |
| MGN.L | Left medial posterior nucleus |
| MV-re.L | Left reuniens (medial ventral) |
| Pf.L | Left parafascicular |
| PuA.L | Left pulvinar anterior nucleus |
| PuI.L | Left pulvinar inferior nucleus |
| PuL.L | Left pulvinar lateral nucleus |
| PuMl.L | Left pulvinar medial lateral nucleus |
| PuMm.L | Left pulvinar medial median nucleus |
| VA.L | Left ventral anterior nucleus |
| VAmc.L | Left ventral anterior magnocellular nucleus |
| VLa.L | Left ventral lateral anterior nucleus |
| VLp.L | Left ventral lateral posterior nucleus |
| VPL.L | Left ventral posterolateral nucleus |
| AV.R | Right anteroventral nucleus |
| CeM.R | Right centromedian nucleus |
| CL.R | Right central lateral nucleus |
| CM.R | Right central medial nucleus |
| LD.R | Right laterodorsal nucleus |
| LGN.R | Right lateral geniculate nucleus |
| LP.R | Right lateral posterior nucleus |
| L-SG.R | Right limitans (suprageniculate) |
| MDl.R | Right mediodorsal lateral magnocellular nucleus |
| MDm.R | Right mediodorsal medial magnocellular nucleus |
| MGN.R | Right medial posterior nucleus |
| MV-re.R | Right reuniens (medial ventral) |
| Pf.R | Right parafascicular |
| PuA.R | Right pulvinar anterior nucleus |
| PuI.R | Right pulvinar inferior nucleus |
| PuL.R | Right pulvinar lateral nucleus |
| PuMl.R | Right pulvinar medial lateral nucleus |
| PuMm.R | Right pulvinar medial median nucleus |
| VA.R | Right ventral anterior nucleus |
| VAmc.R | Right ventral anterior magnocellular nucleus |
| VLa.R | Right ventral lateral anterior nucleus |
| VLp.R | Right ventral lateral posterior nucleus |
| VPL.R | Right ventral posterolateral nucleus |


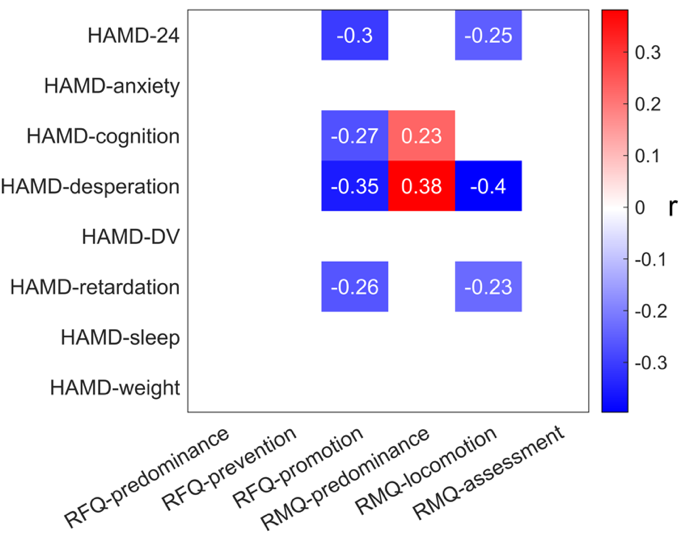


**Figure S1. Behavioral correlation between SR subscales and depressive symptoms in MDD group**. Partial Pearson’s correlation coefficients (*p* < 0.05) between SR subscales and depressive symptoms, with gender, age and education levels as covariates. r, Pearson’s correlation coefficient. All abbreviations can be found in **Table S1** in this Supplement.
